# Crystallization of Polymers under the Influence of an External Force Field

**DOI:** 10.3390/polym13132078

**Published:** 2021-06-24

**Authors:** Rajdeep Singh Payal, Jens-Uwe Sommer

**Affiliations:** 1 Leibniz-Institut für Polymerforschung Dresden, Hohe Strasse 6, 01069 Dresden, Germany; payal@ipfdd.de; 2 Department of Physics, School of Advance Science and Languages, VIT Bhopal University, Kothrikalan, Sehore Madhya Pradesh 466114, India; 3 Institut für Theoretische Physik, Technische Universität Dresden, Zellescher Weg 17, 01062 Dresden, Germany

**Keywords:** polymer crystallization, computer simulations, entanglements

## Abstract

We simulated the crystallization and melting behavior of entangled polymer melts using molecular dynamics where each chain is subject to a force dipole acting on its ends. This mimics the deformation of chains in a flow field but represents a well-defined equilibrium system in the melt state. Under weak extension within the linear response of the chains, the mechanical work done on the system is about two orders of magnitude smaller as compared with the heat of fusion. As a consequence, thermodynamic and simple arguments following the secondary nucleation model predict only small changes of the crystalline phase. By contrast, an increase of the stem length up to a factor of two is observed in our simulations. On the other hand, the lamellar thickening induced by the external force is proportional to the increase of the entanglement length in the melt prior to crystallization as measured by the primitive path method. While the mechanical work done on the system is only a small perturbation for thermodynamics of polymer crystallization, the change of the primitive path is large. This suggests that a strong increase in the lamellar thickness induced, by external deformation, a topological rather than a thermodynamic origin.

## 1. Introduction

Polymer materials undergo crystallization via the partial alignment of their molecular chains. Hereby, molecular chains undergo a transition from a high entropy random coil state to lower entropy partially folded states to form thin lamellar structures on the length scale of nanometers. These crystalline domains coexist with amorphous regions [1,2]. Crystallization is responsible for most of the solid state properties acquired by polymer materials. Despite its great technological importance, polymer crystallization is still an unresolved issue of polymer science. One of the major challenges is to precisely control the semi-crystalline state while polymer crystallization exhibits a strong thermo-mechanical history dependence. Thus, for practical applications, crystalline behavior of the polymers is controlled by varying the processing conditions, e.g., crystallization temperature [3,4], cooling rate [5], or by the application of strain/flow [6,7,8,9,10,11,12]. In general, the thickness of the crystalline lamellae increases (or decreases) with increasing (or decreasing) crystallization temperature. Similarly, higher crystallinity is obtained for a slower cooling rate. Application of shear or strain results in enhanced nucleation and rapid crystallization of polymer materials [9,10,11,12]. Application of shear or strain can also give rise to different crystalline morphologies. In the absence of shear, polymers generally exhibit spherulitical structure. Application of shear can result in shish-kebab morphologies of the crystalline polymer [10,13,14] and fiber formation [15,16,17,18,19]. The microscopic origins of these phenomena are still a subject of debate. This is primarily due to the fact that polymer crystallization leads to non-equilibrium states. For semi-crystalline polymers, there is no thermodynamic phase coexistence but a large gap between melting and crystallization temperatures.

There is increasing evidence that the entanglement density plays a crucial role for the crystallization properties both in quiescent crystallization and under flow conditions. In a recent work by Liu and Yu [20], for instance, it was shown that control over the entanglement state using a complex shear-rate protocol can reveal its central role for the crystallization properties of polycaprolactone (PCL) and blends of PCL/poly(styrene-co-acrylonitrile). Recent coarse-grained simulations of polymer melts subject to a high shear rate and subsequent strong extension of the chains have indicated a significant decrease of the entanglement density [21].

One way to better understand crystallization phenomena in polymers is to study the influence of systematic variations of the melt state on the resultant crystalline structure. In this work, we apply a force dipole to the ends of each chain and investigate the impact of stretching the chains in equilibrium on the crystallization behavior in large scale computer simulations using a systematically coarse-grained model of polyvinyl alcohol (CG-PVA) introduced by Müller-Plathe and coworkers [22], and further developed in our previous studies [23,24]. First, the applied force changes the entropy of the molten state in equilibrium, and thus has a direct consequence on the equilibrium melting temperature, $T_{m}^{0}$, at which an equilibrium phase coexistence between the melt and the perfect crystalline phase is predicted. Second, the application of a force field will change the inter-chain structural properties of the polymer melt, in particular, the degree of entanglement. To quantify the state of entanglement of the polymers at each stage of the crystallization and melting processes, we use a variant of the primitive path analysis (PPA) [25,26]. Our previous work has shown the key role of the primitive path of the polymer chains prior to crystallization for the selection of the lamellar thickness [27,28,29].

The force dipoles mimic in part the effect of a flow field and thus our approach also aims to better understand the strong influence of flow fields on the nucleation and crystalline morphology. In particular, we want to clarify the question: To which extent is the thermodynamics of the deformation of the melt responsible for the changes in the crystallization behavior? While in the case of a flow field, non-equilibrium effects overlay the thermodynamics; in our case, the application of a dipole force field gives rise to a well-defined equilibrium state of the polymer melt. For example, in the case of flow-induced crystallization, the specific work applied by the flow can be measured, which is a non-equilibrium observable depending on dissipation properties of the polymer melt [9]. For the case of force dipoles, the equilibrium free energy of chain stretching can be easily calculated and is directly related with the entropy reduction of the chains in the disordered phase. Therefore, we can clarify if and to what extent the reduction of the entropy in the molten state contributes to the change of the crystalline state. We note that the concept of force dipoles recently has led to the prediction of a force-induced demixing phase transition in fully atomistic simulations of poly(ethyleneoxide) in water [30,31], which has been also found in experiments [32].

The major result of our study is that even weak stretching of the chains enhances crystallization behavior strongly. It results in an increase of the crystallization temperature during cooling and leads to the formation of thicker lamellae at a fixed quenching temperature. In all our simulations, the ratio between the mechanical work done on the chains and the latent heat of fusion is very small (of the order of percent) which makes a simple thermodynamic explanation of the resulting effects unlikely. We exemplify this by applying the classical Lauritzen–Hoffman model [33] to include the effect of external force. On the other hand, the entanglement length as quantified by the primitive path analysis increases strongly with the applied force and offers an alternative explanation of the force-induced crystallization properties.

The rest of this work is structured as follows: In Section 2, we present the thermodynamic analysis of polymer crystallization on weak forces and its consequences on the secondary nucleation model. In Section 3, we present our simulation results including the thermodynamic and topological analysis. Our conclusions are presented in Section 4.

## 2. A Thermodynamic Analysis of the Influence of Stretching on the Crystallization of Polymers

We start by discussing the most simple thermodynamic consequences of the applied dipole force on the polymer system. In particular, we consider the changes of the phase equilibrium and we apply the main concepts of the secondary nucleation model to derive a few consequences on the crystallization properties within this model.

**Figure 1:**
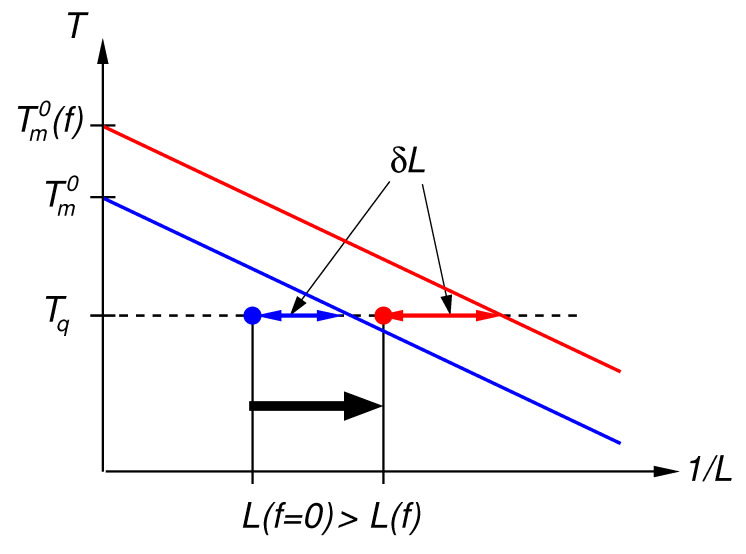
Sketch of the state diagram. The x-axis denotes the inverse stem length and the y-axis represents the temperature. The equilibrium melting temperature without perturbation by the external force is given by $T_{m}^{0}$. The force field leads to a shift towards higher equilibrium melting temperatures, $T_{m}^{0}\left(f\right)$. The Gibbs–Thomson melting point depression due to the surface tension of the lamellae is represented by straight lines in this representation. Upon quenching, in order to form a stable-growing state, the selected lamellar thickness has to be larger then the stability limit which can be represented by offset of δL (double arrows) with respect to the Gibbs–Thomson line. The crystallization states are indicated by the full circles. The expected shift in the lamellar thickness due to application of force is indicated by the black arrow.

The primary thermodynamic effect of the applied force is a shift in the equilibrium melting temperature, $T_{m}^{0},$ of the polymer towards a higher value, $T_{m}^{0}\left(f\right)$, as sketched in Figure 1. Using a first order thermodynamic expansion for the phase equilibrium, the latter is given by the relation

(1) $$T_{m}^{0}\left(f\right)=T_{m}^{0}\left(1+\eta \right)\;\;,$$

with the (small) parameter

(2) $$\eta =\frac{\Delta G}{H_{f}^{0}}\;\;,$$

which denotes the ratio between the change of the elastic free energy, ΔG, of the polymers in the melt at the equilibrium melting temperature and the latent heat of fusion $H_{f}^{0}$. In this calculation (to the leading order in η), it is assumed that $H_{f}^{0}$ is the heat of fusion of the ideal polymer crystal as it would be formed in thermodynamic equilibrium with the melt state. The derivation of the above equation is based on the Gibbs free energy balance at the equilibrium melting temperature: $H_{f}-S_{f}T_{m}^{0}\left(f\right)+\Delta G=0$, with the approximation of a small perturbation by ΔG. The latter justifies the approximations of $H_{f}≃H_{f}^{0}$ and $S_{f}≃H_{f}^{0}/T_{m}^{0}$, which result from the phase equilibrium without the perturbation by the external force. We note that this derivation is in full analogy to the calculation of melting point depression due to the Gibbs–Thomson-effect [33,34].

As we will show in our simulations, η is a very small parameter, typically of the order of $10^{-2}$, see Table 1. This is quite intuitive by making a simple estimation: The heat of fusion needs to be in the order of a few $k_{B}T$ ($k_{B}$—Boltzmann constant) per Kuhn segment in order to freeze out the rotation degrees of freedom of each segment. On the other hand, the work of deformation in the Gaussian region of long chains is typically of the order of $10\;k_{B}T$ per chain. This can be understood by the Gaussian elastic energy per chain at a given extension ratio, λ, of the chain: $\Delta G=\frac{3}{2}k_{B}T\lambda^{2}$. Given a chain of 1000 monomers, this results in the above noted order of magnitude for η. Very high deformations, typically well beyond the Gaussian range, are necessary to obtain much larger values for ΔG. We note that much higher stretching energies in the order of the energy of a chemical bond, i.e., about $100\;k_{B}T$ (which is still 10 times lower as compared with the heat of fusion of the chain with 1000 units) can break the polymers; thus, values of η of the order unity are non-physical. As an example, PVA has an equilibrium melting point of about $T_{m}^{0}=500$ K; thus, we would obtain a mild shift to about $T_{m}^{0}\left(f\right)≃505$ K for small forces.

Next, we consider the effective melting point of thin lamellae of thickness *L*. The standard Gibbs–Thomson argument applied to this geometry, see Ref. [33], gives

(3) $$T_{m}=T_{m}^{0}\left(f\right)\left(1-\frac{2\sigma_{f}v}{H_{f}^{0}}L^{-1}\right)\;\;.$$

Here, $\sigma_{f}$ defines the surface tension of the fold surface of the lamellar crystal and *v* denotes the specific volume of the monomer, given $H_{f}^{0}$ in molar units of monomers. This equation is sketched as blue and red lines in Figure 1. The applied force, if not extremely strong as we will assume throughout this work, will not deform the crystalline phase and thus should not change the latent heat of fusion. Furthermore, also the energy of the folding surface should not be influenced much by the force. The latter is given by the free energy of small folds and a contribution from molecular surface tension due to the solid–liquid interface, see for instance Ref. [35] where the equilibrium properties of folded chain crystals are calculated. Thus, the melting line of lamellae in the presence of an external force is just shifted to higher temperatures, as sketched by the red line in Figure 1.

In order to predict the lamellar thickness at a given crystallization temperature, $T_{q}$, lamellae have to be formed which are thicker then the Gibbs–Thomson limit. Thus, we have to claim

(4) $$L=L_{0}+\delta L\;\;,$$

with an offset of δL. In the standard Lauritzen–Hoffman secondary nucleation model [33], δL is given by

(5) $$\delta L≃\frac{k_{B}T_{q}}{2b\sigma }\;\;,$$

where *b* is the thickness of the stem in the growth direction and σ denotes the surface tension at the growth front. If we follow the argument given in Ref. [36], a small reduction of σ due to the entropy reduction of the chains under force may be expected which, again, is of the order of η. Thus, we arrive at the selected thickness $L(f,T_{q})$ as sketched in Figure 1. Here, $T_{q}$ denotes the temperature of the isothermal quench into the crystalline phase. Note that the shift of the inverse length (1/L) is larger on the right, i.e., towards smaller thicknesses. The predicted shift in the lamellar thickness, $L\left(f,T_{q}\right)-L\left(0,T_{q}\right)$ is a bit smaller as compared with the parallel shift of the melting lines in the x-direction and is given by the arrow in the figure. We stress again that possible corrections to the surface tensions in this model need to be expressed in terms of the small parameter η.

We can conclude that the effect of the applied force leads to thinner lamellae at the same crystallization temperature, apart from a possibly small correction due to the entropic reduction of σ [36]. From the viewpoint of the secondary nucleation theory, this result is intuitive: The shift of the equilibrium melting temperature due to the entropy reduction of the melt state leads to a stronger effective under-cooling at a given crystallization temperature and thus to quicker growth and thinner lamellae. Under the consideration that a weak force (η≪1) does not influence the kinetics of the crystallization process on the scale of monomers, indeed the application of the force can be mapped to equivalent system with a higher under-cooling only.

## 3. Simulation Results

Involved time and length scales imposes major obstacles in simulating polymer crystallization at fully atomistic scale. Thus, coarse grained models are used to substantially reduce the computational cost. The coarse grained model developed by Müller-Plathe and coworkers [22] for the polyvinyl alcohol (CG-PVA) is suitable to represent linear and flexible polymers. The advantage of the CG-PVA model is its computational efficiency. In particular, we implemented an extension of the LAMMPS package which provides a high parallel performance of the code [23]. Since we are interested in generic properties of polymer crystallization (such as the role of entanglements), the choice of the particular model is not of primary importance. We note that it is of utmost importance to reach long time (crystallization dynamics) and length scales (highly entangled polymers).

### 3.1. Application of Force Dipoles to Melt State

**Figure 2:**
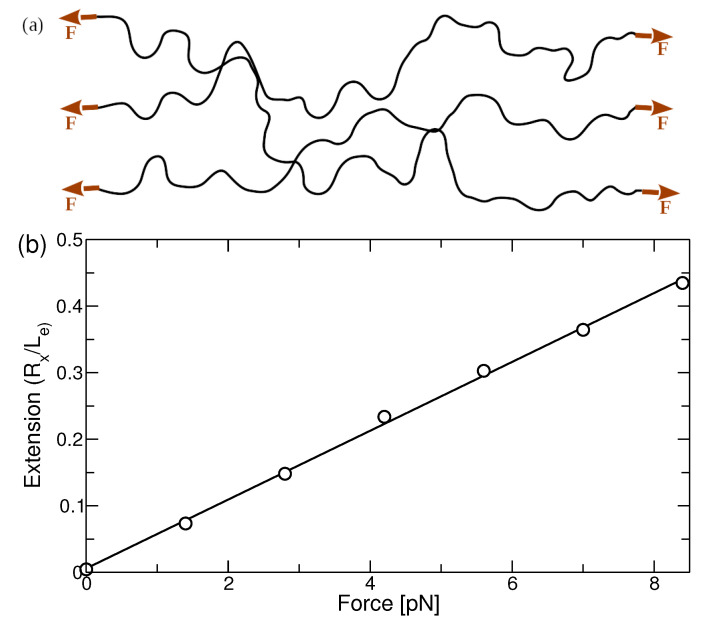
(**a**) Schematic for the application of force dipole. (**b**) Extension ratio of the polymer chains along the direction of the force in the reference state. In this work, only weak forces in the linear stretching regime are used.

Equal and opposite forces (force dipoles) were applied to the ends of the polymer chains in the x-direction, see Figure 2a. This allows the chains to freely fluctuate and diffuse but imposes an elastic stretching of each chain. This concept has been developed earlier to study a stretching-induced phase transition in PEO-water-solutions [30,31]. Simulations were carried out for seven different systems with varying strength of applied force, see Table 1. Each system consists of 1000 polymer chains with 1000 monomers per chain. Simulations were performed using the LAMMPS [37] code. For all the simulations, pressure of the system was maintained at 1 atm. All the systems were first equilibrated in the melt state at 550 K for 3.5 µs. We note that this temperature is about 25 K above the extrapolated equilibrium melting temperature of CG-PVA in our simulations. We denote this as the reference state in the following. All molar units are given in mole of monomers.

The elongation of flexible polymer chains with respect to the dipole force in the melt state can be described by the Langevin function:

(6) $$\frac{R_{x}}{L_{e}}=\coth\left(\frac{fl_{k}}{k_{B}T}\right)-\frac{k_{B}T}{fl_{k}}≃\frac{fl_{k}}{3k_{B}T}\;\;\;\mathrm{for}\;\;\;fl_{k}\ll k_{b}T\;\;,$$

where $R_{x}$, $L_{e}$, and $l_{k}$ are the average end-to-end distance along the direction of applied force, the full extension length of the chains, and Kuhn length, respectively. In the low-force regime (Gaussian regime), the force-extension relation is given by a linear function, see rhs of Equation (6). Figure 2b shows the simulation results for the extension ratio as a function of the applied force. The data follow a linear function suggesting that polymer chains do not deviate from Gaussian behavior in the range of forces applied in this work. The value of the Kuhn length, $l_{k}=12.2$ Å, obtained from the force extension curve is in good agreement with the previous calculations [38].

**Figure 3:**
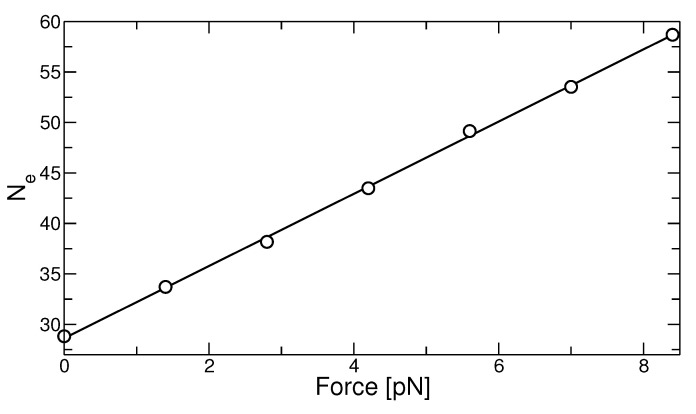
Entanglement length in units of monomers obtained from PPA analysis as a function of applied force.

The applied force has also consequences on the topological state of the polymer melt. The latter can be characterized by the entanglement length, $N_{e}$, as obtained by the primitive path analysis (PPA) in our work. This method was originally proposed by Everaers et al. [25] and we use the implementation described in our previous work, see Refs. [26,27]. Alternatively, $N_{e}$ can be calculated using the ‘Z1’ analysis proposed by Kröger [39]. Both methods yield similar results with a small difference in the values of $N_{e}$ [28]. In Figure 3, we display the result for $N_{e}$ in the reference state as a function of the applied force. The stretching of the chains lead to partial disentanglement of the polymers, and also $N_{e}$ increases linearly with increasing force. The influence on the topological state is essential: The entanglement length as estimated by the PPA increases up to a factor of two. A comparable result has been obtained in the previous work of Sliozberg et al. [21] simulating uniaxial extension of a coarse-grained PE melt.

### 3.2. The Crystallization of Polymer Melts under Force

**Figure 4:**
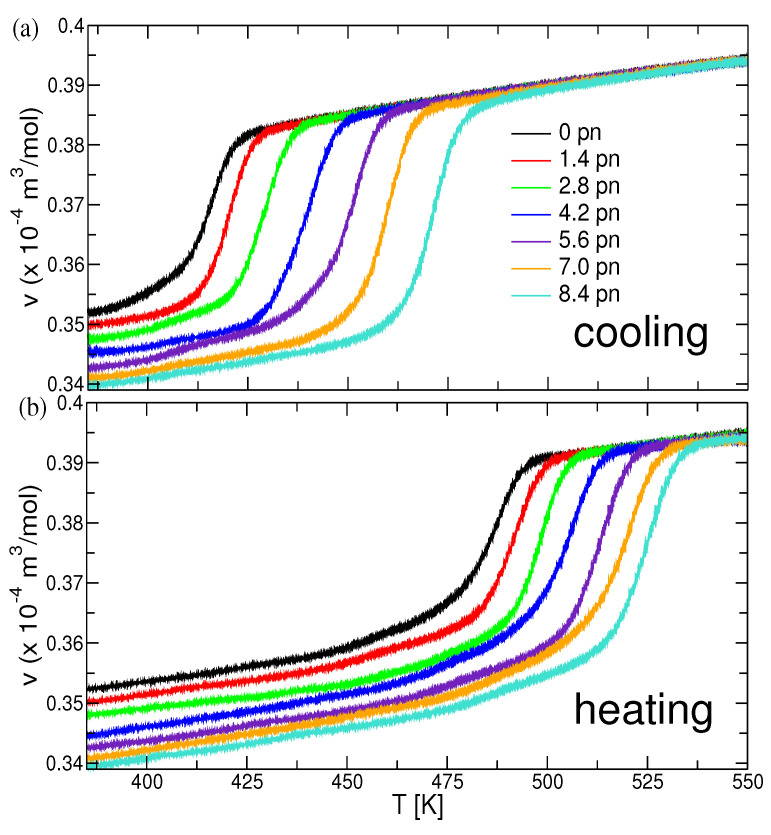
Specific volume as a function of temperature during (**a**) cooling and (**b**) heating cycles.

Equilibrated systems were cooled from 550 K to 380 K with a cooling rate of 48.6 Kµs${}^{-1}$. Similar to the previously reported results [26], the crystallization process is dominated by homogeneous nucleation. Crystallized systems obtained after cooling were again heated to 550 K with heating rate the same as cooling rate. The influence of the applied force on the crystallization process is evident from the cooling and heating curves shown in Figure 4a,b, respectively. Sharp decrease or increase in the specific volume (*v*) corresponds to the partial crystallization, or melting of the system. Crystallization and melting temperatures were calculated by determining the inflection points of v(T).

**Figure 5:**
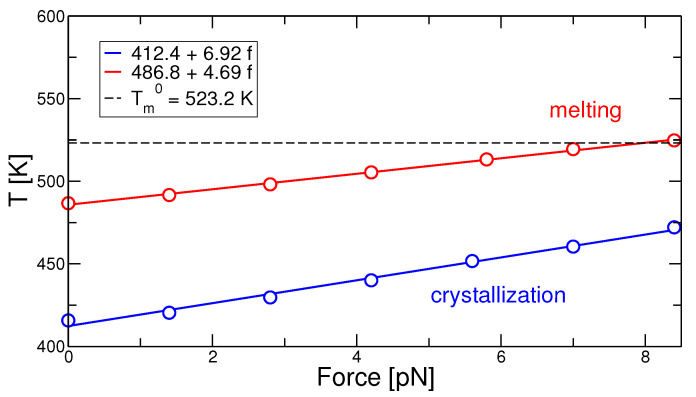
Crystallization and melting temperature as a function of the applied force taken from the inflection point of the heating/cooling data of Figure 4. The dashed lines indicate the extrapolated equilibrium melting temperature, $T_{m}^{0}≃523.2$ K.

Crystallization and melting temperatures are shown in Figure 5: Systems under higher force crystallize and melt at higher temperatures, respectively. The crystallization temperature exhibits a slightly stronger dependence on the applied force as compared to the melting temperature. This large hysteresis (gap) between crystallization and melting is characteristic of semi-crystalline polymers. The increase in the crystallization and melting temperature of polymers due to application of shear or strain has been well documented in experimental studies, see Ref. [40]. Note that the melting point at the highest force is above the equilibrium melting temperature for the quiescent polymer, $T_{m}\left(f>6.5\;pN\right)>T_{m}^{0}≃523$ K [29].

**Figure 6:**
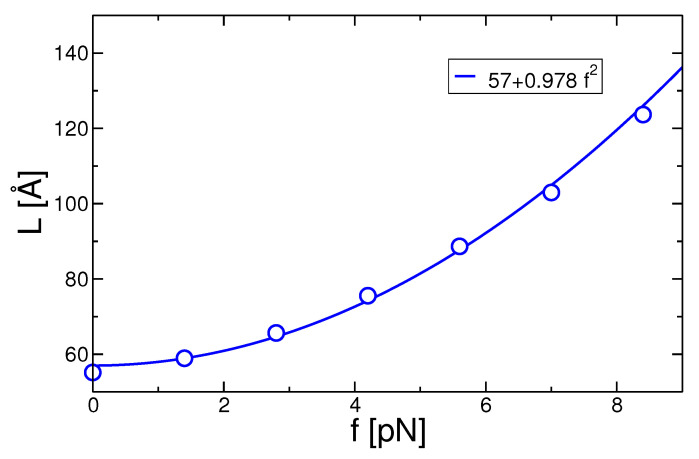
Lamellar thickness as a function of the applied force. Simulation data are open symbols.

The lamellar thickness, or stem length, *d*, is determined by calculating the sequence of successive trans–trans segments. Stem length of d≥15 (in monomer units) was set as the cutoff for crystalline lamellar in order to not account for spontaneous alignment of segments as it occurs in the melt state too. Details are given in our previous work [41]. We have extended the established methodology to take into account defects embedded in the crystalline lamellae. These defects arise due to the presence of small non-trans sequences up to 5 coarse-grained units between two co-aligned crystalline segments and occur more frequently in strongly under-cooled systems. In order to calculate the correct stem length, first we check the orientation of the two crystalline sequences which are connected by the defect. If the two sequences are parallel, it is considered as a single stem containing a defect. In Figure 6, we show the results for the lamellar thickness as a function of the applied force. The increase of the lamellar thickness is approximately quadratic with the force; thus, it is proportional to the work applied to the chains.

### 3.3. Thermodynamic Analysis and Instantaneous Quenching Simulations

The results for continuous cooling protocols do not allow for a direct comparison with the thermodynamic analysis introduced in Section 2. Under these conditions, the crystallization sets in earlier (at higher temperatures) for higher applied forces and leads to thicker lamellae. It is not easily possible to discriminate between the effect of higher crystallization temperature and the direct influence of force on the lamellar thickness. Therefore, next we apply an instantaneous quenching protocol which sets a definite reference state (fixed quenching temperature) for comparison with thermodynamic analysis. We first analyze the essential thermodynamic parameters.

**Figure 7:**
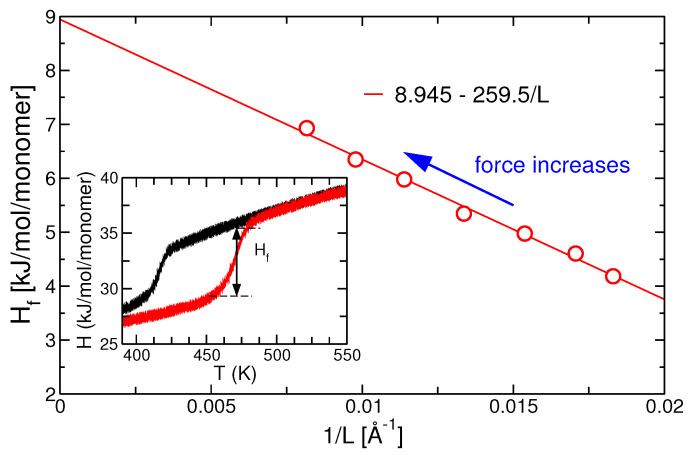
Enthalpy of fusion as a function of the inverse stem length. Different data points correspond to different forces. The inset displays the enthalpy as a function of temperature during a cooling/heating cycle for zero force.

**Figure 8:**
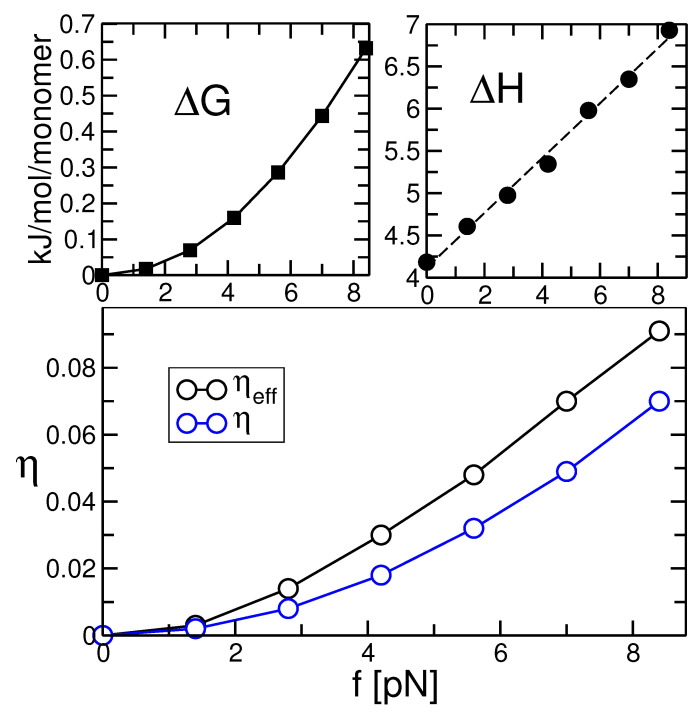
Gibbs free energy of stretching (left upper plot) and enthalpy of fusion (right upper plot) as a function of force in pN. The ratio, η, between the free energy of stretching and the heat of fusion, see Equation (2), as well as the effective ratio, $\eta_{eff}$, using the value of $H_{f}$ for the given force, are shown in the lower plot.

The enthalpy of fusion, $H_{f}$, is obtained by taking the difference of the enthalpy at the end and at the beginning of the melting process as indicated in the inset of Figure 7. Onset and completion of the crystallization or melting was determined by the deviation of the enthalpy curve from its linear behavior before and after the crystallization or melting. We note that $H_{f}$ is the apparent enthalpy of fusion in the semi-crystalline system. With increasing force, $H_{f}$ increases as shown in Figure 7 and upper right plot of Figure 8. When plotted against the inverse lamellar thickness, one can extrapolate the heat of fusion of the infinite polymer crystal, $H_{f}^{0}≃8.945$ kJ/mol. We note that at T=500 K we obtain $N_{A}k_{B}T≃4$ kJ. Thus, the heat of fusion per monomer is between $1\;\mathrm{and}\;2\;k_{B}T$.

In Figure 8, we compare the Gibbs free energy of stretching, ΔG, with the heat of fusion, $H_{f}$. Particularly interesting is the ratio of the two energies, $\eta_{eff}$, as well as the ratio between ΔG and the extrapolated equilibrium heat of fusion, $H_{f}^{0}$, denoted by η, see Equation (2). Both numbers are displayed in the lower plot of Figure 8. As can be seen, η and $\eta_{eff}$ are in the order of percent, even of a fraction of percent for low forces. According to our calculation in Section 2, this means that all shifts in the equilibrium melting temperature and of the expected lamellar thickness (according to the secondary nucleation model) should be very small. The essential thermodynamic values are listed in Table 1.

As we have concluded in Section 2, an increase in ΔG leads to an increase in the equilibrium melting temperature of the system according to Equation (1). Assuming that neither the surface tension of the fold surface nor the value of δL, see Equation (5), are influenced by the very small parameter η, we expect that at a given crystallization temperature, $T_{q}$, the lamellar thickness should decrease with increasing force according to

(7) $$L\left(f\right)-\delta L∼\frac{1}{T_{m}^{0}\left(f\right)-T_{q}}\;\;.$$

**Figure 9:**
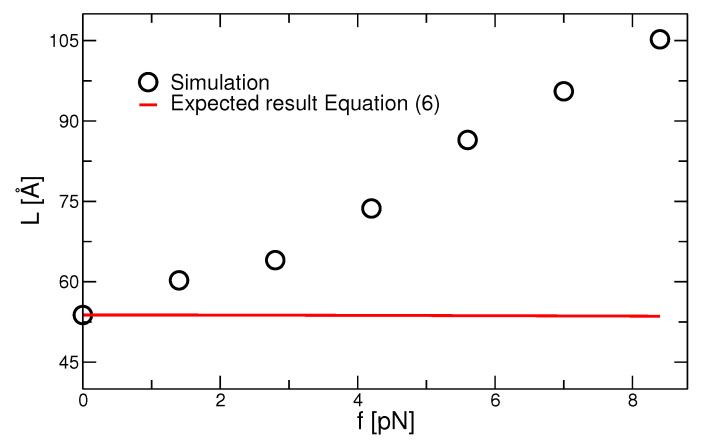
Simulation results for the stem length formed during isothermal crystallization performed by quenching the melt to $T_{q}=430$ K, black symbols. The theoretical prediction as discussed in the text is given by the red line according to Equation (7). The symbols indicate the values of the force used in the simulation.

To test this prediction, we simulated a protocol, which we call QC, where the temperature of the equilibrated melt is set instantaneously to the crystallization temperature $T_{q}$. For a comparison of the different protocols in previous work, see also Ref. [29]. Then, the system was allowed to equilibrate isothermally for 3.2 µs. Figure 9 shows the comparison of the stem lengths obtained from simulation as a function of the applied forces. Contrary to the theoretical prediction, the stem length increases with increasing force as it was also the case for the constant cooling protocol. In the figure, we also plot the expected lamellar thickness, L(f), according to Equation (7). Here, we take the values of $T_{m}^{0}$ as obtained from Equation (1) using the computed values of η, see last column in Table 1. The prefactor in Equation (7) is fixed by taking the stem length at f=0.

We note not only the opposite trend of the change in lamellar thickness with force, but also the absolute value: According to the thermodynamic arguments, the expected change is of the order of the value of η, i.e., in the range of percent. In contrast, the observed stem length changes by more then 100 percent over the range of the forces. At the same time, the crystal growth rate increases with force (data not shown). We note that a simultaneous increase in stem length and growth rate is in contradiction with the secondary nucleation model if the mobility of the polymer would not increased dramatically at the same time.

### 3.4. Entanglements and Stem Length Selection under Force

We have shown in our previous work that the selection of stem lengths can be directly related to the state of entanglement of the polymers in the under-cooled melt before the onset of crystallization. A similar conclusion has been drawn from experimental studies [42,43] which showed that the entanglement state of under-cooled melt controls the crystallization behavior. In particular, these works have demonstrated that the nucleation rate decreases with increasing entanglement density in a non-equilibrium melt which was prepared by melting extended chain crystals. As we have demonstrated above, the application of force increases the entanglement length in the reference (melt) state up to a factor of two in our simulations, see Figure 3. This is in agreement with previous simulations in uniaxially deformed melts [21]. Therefore, the force-induced reduction of the primitive path offers an alternative explanation for the strong increase of the stem length at isothermal conditions.

**Figure 10:**
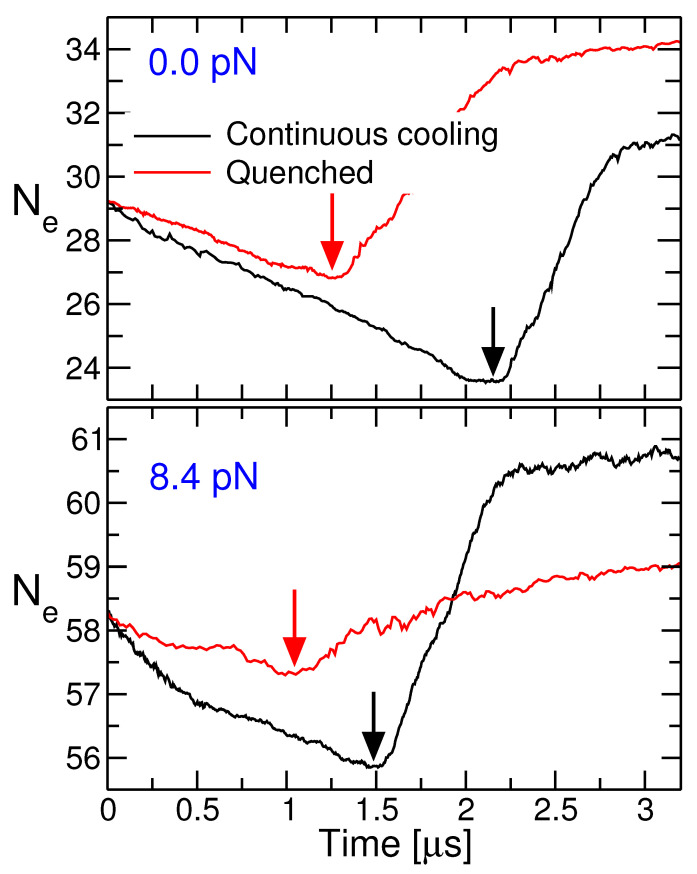
Change of the entanglement length during crystallization vs. time. Without force (**upper plot**) and for f=8.4pN (**lower plot**). The arrows indicate the onset of crystallization in the under-cooled melt. Data for the quenched states are taken at 430 K.

Figure 10 shows the time evolution of average entanglement length during cooling. For both cooling protocols, initially $N_{e}$ decrease upon cooling. This can be attributed to the stiffening of polymer chains upon cooling; for an in depth discussion, see Ref. [26]. Increase in $N_{e}$ after the onset of crystallization suggests that crystallization occurs with partial disentanglement of the chains. The lapse of time before the onset of crystallization is significantly shorter for the QC protocol. Furthermore, $N_{e}$ exhibit less reduction for QC protocol. Here, the sudden change in temperature partially inhibits the system to readjust its entanglement density [29]. A strong force dependence of $N_{e}$ is conserved upon cooling: For both protocols, $N_{e}$ decreases for a shorter period of time and decrement in $N_{e}$ reduces with increasing force.

**Figure 11:**
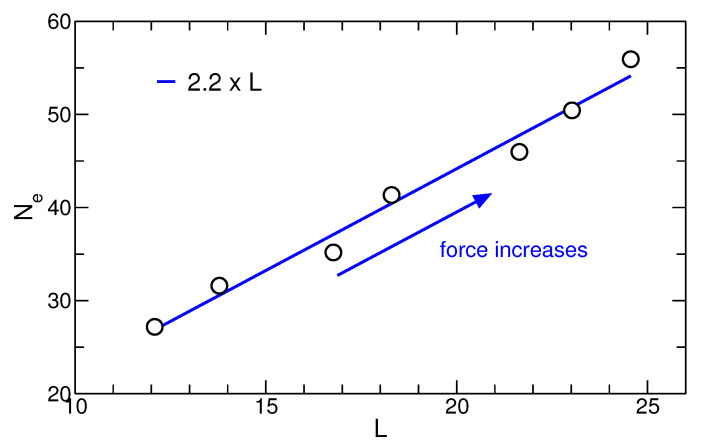
Entanglement length in the under-cooled state at the onset of crystallization vs. stem length in monomer units. The ratio of 2.2 between the two length scales is preserved upon application of force.

In Figure 11, we plot the value of $N_{e}$ of the under-cooled melt (at the onset of crystallization) as a function of the stem length for the QC protocol. Within the range of our simulation parameters, a linear relation between selected stem length and entanglement length in the under-cooled state is preserved:

(8) $$N_{e}=f\cdot L\;\;\mathrm{with}\;\;f≃2.2\;\;.$$

The folding ratio of f≃2.2 is very close to the value obtained for simulations of diluted polymer melts [28].

## 4. Conclusions

Application of a force-dipole to individual polymer chains in the melt state mimics the stretching effect of a flow field. However, the resulting stretched conformations are in a well-defined equilibrium state which allows for the application of equilibrium thermodynamic arguments. We have shown that small forces in the range of pN can change dramatically the properties of the resulting crystalline state: Crystallization is accelerated and longer stems are formed at fixed under-cooling. While our observations qualitatively agree with those for flow-induced crystallization, they are in some contrast to the expectation from the secondary nucleation model. First of all, the applied force shifts the equilibrium melting temperature to higher values due to the reduction of entropy in the melt state. By assuming that other parameters of the secondary nucleation model remain unchanged, this implies smaller stem lengths because of the higher effective under-cooling. Moreover, the expected changes due to the thermodynamic effect are very small. We have defined the ratio, η, between the elastic energy due to stretching (typically several $k_{B}T$ per chain) and the latent heat of fusion (typically several $k_{B}T$ per monomer) which enters the thermodynamic calculations. In our simulations, η is of the order of $10^{-2}$. An increase of the stem length up to a factor of two is, therefore, irrespective of the sign of the change, very difficult to explain. In short, viewed from the thermodynamic perspective, the weakly stretched chains correspond to a melt with a slightly higher equilibrium melting point, and thus at a given crystallization temperature this corresponds to a slightly more under-cooled system. We note that the stretching of chains might impact the fold surface tensions as well as the nucleation barrier via a decrease on the lateral surface tension. However, the free energy impact due to stretching should be again of the order of η because the reference energies per monomer unit in the crystalline state are of the order $k_{B}T$. Thus, a dramatic decrease of the nucleation barrier should not be expected.

Nevertheless, our simple analysis cannot provide a final answer about a possible kinetic origin of the force-induced lamellar thickening. In order to understand the impact of force on primary nucleation, single chain crystallization under the influence of a stretching force could be studied as an interesting model system. Here, analytical results can be obtained [35,44].

Independently of these arguments, the second aspect of application of forces is a disentanglement of the long polymer chains in the melt state. The relative increase of the entanglement length due to the applied force, as defined by the primitive path analysis, is almost two orders of magnitude larger than the relative increase of the equilibrium melting temperature. Thus, the topological state is a possible candidate to explain the large changes in the crystallization behavior. Indeed, we find a linear relation between the stem length and the entanglement length in the under-cooled melt prior to crystallization.

Our results suggest that the change in the topological state due to an external force field can be the key to understand the dramatic changes in the force-induced crystallization behavior which involves an increase of stem length, a faster onset of crystallization during cooling, and a larger crystalline growth rate.

## Figures and Tables

**Table 1:** Thermodynamic parameters for different strengths of the applied force. The ratio η is defined in Equation (2). The effective ratio is given by $\eta_{eff}=\Delta G/H_{f}$. The extrapolated equilibrium melting temperature has been calculated based on Equation (1). The extrapolated equilibrium heat of fusion is given by $H_{f}^{0}=8.945$ kJ/mol, see Figure 7.

Force	$H_{f}$	ΔG	$\eta_{\mathrm{eff}}$	η	$T_{m}^{0}\left(f\right)$
(pN)	(kJ/mol/Monomer)	(kJ/mol/Monomer)	(%)	(%)	K
0.0	4.183	0.000	0.0	0.0	523.2
1.4	4.605	0.017	0.3	0.2	524.2
2.8	4.975	0.069	1.4	0.8	527.4
4.2	5.345	0.159	3.0	1.8	532.6
5.6	5.979	0.286	4.8	3.2	539.9
7.0	6.349	0.443	7.0	4.9	548.8
8.4	6.929	0.632	9.1	7.0	559.8

## Data Availability

Not applicable.
